# Frailty transitions and associated clinical outcomes in patients with stable COPD: A longitudinal study

**DOI:** 10.1371/journal.pone.0230116

**Published:** 2020-04-03

**Authors:** Roberto Bernabeu-Mora, Silvana Loana Oliveira-Sousa, Mª Piedad Sánchez-Martínez, Jose Antonio García-Vidal, Mariano Gacto-Sánchez, Francesc Medina-Mirapeix

**Affiliations:** 1 Department of Pneumology, Hospital General Universitario JM Morales Meseguer, Murcia, Spain; 2 Research Group Fisioterapia y Discapacidad, Instituto Murciano de Investigación Biosanitaria-Virgen de La Arrixaca (IMIB), Murcia, Spain; 3 Department of Physical Therapy, University of Murcia, Murcia, Spain; 4 Department of Physical Therapy, EUSES University School, University of Girona, Girona, Spain; Sao Paulo State University (UNESP), BRAZIL

## Abstract

**Background:**

Although frailty is a frequent occurrence in chronic obstructive pulmonary disease (COPD) patients, evidence on the frequency of frailty transition is scarce.

**Aims:**

The present study aimed to describe the frailty status transition rates over a 2-year period and their associated clinical outcomes in stable COPD patients, and to determine predictors of improvement in frailty status.

**Methods:**

We prospectively included 119 patients with stable COPD (mean age ± SD, 66.9 ± 7.9 years) over a follow-up period of 2 years. Frailty was assessed using the Fried criteria (unintentional weight loss, weakness, exhaustion, low activity level, and slow walking speed). Several demographic, clinical, and health-related variables were measured. We calculated the rates for each of the frailty transitions (no change, improvement, or worsening) between baseline and 2 years. Outcomes were compared using one-way analysis of variance and predictors of improvement were identified in multivariate logistic regression.

**Results:**

After 2 years of follow-up, 21 (17.6%) patients had an improved frailty status, 14 (11.7%) had worsened, and 84 (70.5%) had maintained the same frailty status. The worsening group (*vs* no change group) had greater dyspnea (*p* = 0.013) and disability (*p* = 0.036) and lower handgrip strength (*p* = 0.001). In contrast, the improved group (*vs* no change group) had greater handgrip (*p*<0.001) and quadriceps strength (*p* = 0.032). Furthermore, the improved group had greater handgrip strength (*p*<0.001), quadriceps strength (*p* = 0.003), physical activity (*p* = 0.008), and lower disability (*p* = 0.019) than the worsening group. Additionally, we determined that the 5STS test (≤ 13.6s) and exacerbations (≥ 2) were independent predictors for improvement in frailty status [adjusted OR 9.46, *p* = 0.058 and adjusted OR 0.12, *p* = 0.026, respectively].

**Conclusions:**

Frailty is a dynamic process for approximately one-third of patients with stable COPD and transitions in frailty status are associated with significant changes in clinical outcomes. The 5STS and exacerbations were independent predictors of improvement in frailty status.

## Introduction

Frailty is a geriatric syndrome characterized by multisystem decline leading to reduced functional reserve and increased vulnerability to adverse outcomes [1]. A validated phenotype of physical frailty has been shown to predict incident and worsening disability, hospitalizations, and mortality [2,3]. According to this well-established and validated model, frailty comprises five individual physical characteristics: unintentional weight loss, low physical activity, exhaustion, slow walking speed, and low grip strength. The presence of one or more characteristics determines the patient’s frailty status (i.e., pre-frailty or frailty) [2,3].

Although frailty has been traditionally associated with age, patients with chronic obstructive pulmonary disease (COPD) also often present with extrapulmonary clinical manifestations frequently associated with frailty, such as physical inactivity, muscle weakness, anorexia, osteoporosis, and fatigue [4]. Consequently, approximately 20% of them are frail and 56% are pre-frail [5].

Studies conducted among community-dwelling older adults suggest that a patient’s frailty status has a dynamic nature over time, with transitions between frailty status in both directions (i.e., improvement or worsening of the initial status) [6–13]. In a recent systematic review, Kojima et al. [12] reported rates of worsening that are higher than rates of improvement (29.1% vs. 13.7%). In addition, one-step transitions between adjacent states (e.g., from frail to pre-frail status) have been reported to be more common than two-step transitions (e.g., from frail to no frail) [10–12].

Despite the high prevalence of frailty in COPD patients [14], evidence on the frequency of frailty transitions over time under a particular intervention is scarce. To the best of our knowledge, solely one study, which was carried out in a pulmonary rehabilitation setting, reported that 60% of frail patients improved their initial status after 6 months of participating in a physical rehabilitation program [15]. This study also demonstrated that the final frailty status at the end of the rehabilitation program was associated with relevant changes in clinical outcomes (e.g., quadriceps strength) [15]. However, these transition rates and associated changes in clinical outcomes cannot be extrapolated to other contexts, such as medical follow-up visits.

The objectives of the present study were to describe transition rates between frailty status over a 2-year period and clinical outcomes associated with frailty transitions in stable COPD patients participating in annual medical follow-up visits. We also sought to determine predictors of improvement in frailty status.

## Materials and methods

### Design and participants

Patients with stable COPD were prospectively recruited from an outpatient pulmonary service at Morales Meseguer Hospital, Murcia, Spain. The diagnosis of COPD and the stage of disease were based on the Global Initiative for Chronic Obstructive Lung Disease (GOLD) guidelines. Patients with COPD who were 40–80 years of age were eligible to participate when they had a post-bronchodilator ratio of forced expiratory volume in 1 second (FEV1) to forced vital capacity (FVC) < 0.70. Patients were excluded if they had an unstable cardiac condition within the 4 months prior to the study, cognitive deterioration, or a limitation in walking. During a 1-year period, a consecutive sample of eligible patients was identified from the hospital registry from among all patients with stable COPD who attended their follow-up visits. A pulmonary physician assessed the eligibility criteria for recruitment. Patients underwent control medical examinations at baseline and annually for 2 years. The institutional review board of Morales Meseguer Hospital approved the study protocol (approval number: EST-35/13), and written informed consent was obtained from all study participants prior to study entry.

### Frailty transition assessment

Frailty transitions were evaluated through changes in the frailty status between baseline and 2 years of follow-up. Frailty status was defined according to Frield's phenotype [2], which includes five criteria: 1) unintentional weight loss ≥ 4.6 kg of body weight in the last year according to the medical record; 2) low physical activity identified by the Spanish Short Version of the Minnesota Leisure Time Physical Activity Questionnaire (VREM) [16] (in the lower quintile adjusted by sex); 3) exhaustion as identified by two questions from the CES-D scale [17] (a positive answer to one of the two questions); 4) slow walking speed as assessed by the 4-m gait-speed [18] (lower than the 20^th^ percentile and adjusted for sex and height); and 5) low grip strength as assessed by a handgrip dynamometer [19] (less than 20^th^ percentile, adjusted for sex and body mass index [BMI]). Participants were classified as frail if they met three or more of these criteria, as pre-frail if they met one or two of the criteria, and as non-frail if they met none of the criteria.

Frailty transitions were categorized into three groups: no change (patients who maintained the initial status at 2 years), improvement (patients who changed their status from frail to pre- or non-frail or from pre-frail to non-frail), and worsening (patients who changed from non-frail to pre-frail or frail status or from pre-frail to frail).

### Potential associated clinical outcomes and other variables

Several clinical variables were selected from the literature based on their potential association with frailty. These variables were classified into three domains: pulmonary-related, physical performance, and self-perceived health variables. In addition, quantitative changes in three frailty criteria variables (i.e., handgrip strength, metabolic equivalent tasks [METs], and 4 m gait speed test [4MGS]) were also used as clinical outcomes.

The pulmonary-related domain included the predicted percentage of forced expiratory volume in 1s (%FEV_1_) and the percentage of forced vital capacity (%FVC). The physical performance domain included the 6-minute walking test (6MWT), the short physical performance battery (SPPB) [20], the five-times sit-to-stand test (5STS) [21], and quadriceps strength measured using a hand-held dynamometer [19]. The self-perceived health domain was related to the perception of dyspnea-associated activity according to the modified Medical Research Council (mMRC) dyspnea scale [22], health status measured by the COPD Assessment Test (CAT) [23] and one question from the 36-item Short Form Health Survey Scale from the Medical Outcomes Study [24], and the perception of disability measured by a self-reported mobility questionnaire [25].

Demographic variables included sex, age, education level, and marital status. Other health-related variables included BMI, smoking pack-years, comorbidities (age-adjusted Charlson index), and total number of exacerbations in the previous year (both moderate and serious). All of these characteristics were obtained from the patient’s clinical history.

### Statistical analysis

Data on the sociodemographic and clinical characteristics of patients at baseline are presented as proportions or means (standard deviation [SD]). We used Pearson chi-squared or Fisher’s exact test for normally distributed data, and one-way analysis of variance or Kruskal-Wallis for non-normally distributed data to compare the main characteristics between patients who remained in the study and those who were lost to follow-up. We calculated the percentage of frailty status at baseline and 2 years, and the rates of frailty transitions by groups of initial frailty status. For outcome measures, we calculated the average difference between baseline and 2 years and compared between groups of frailty transition (no change, improvement, and worsening) using one-way analysis of variance with Bonferroni correction applied to a post hoc pairwise comparison. We also used this test to examine differences in the variable outcomes between the groups at baseline. Chi-square and t-test tests were used to compare proportions and means of covariables between the improvement and worsening groups. Univariate and multivariate logistic regression (forward method) was also used to examine the association between covariates and patterns of change (improvement vs worsening) in frailty status. Candidate variables for inclusion in the multivariate model were those with p< 0.20. Only those with p <0.10 were retained in the final model. The goodness-of-fit of the multivariate model was assessed through the Hosmer-Lemeshow test. P-values < 0.05 were considered significant in all analyses.

## Results

A total of 137 patients were included at baseline after excluding 10 patients due to unstable cardiac condition [5], cognitive deterioration [3], or being unable to walk [2]. A total of 119 (86.9%) patients (104 males and 15 females) remained at the end of the 2-year follow-up. Participants missed the follow-up visits for the following reasons: six (4.3%) passed away, eight (5.8%) dropped out because of lung cancer and four (3%) chose not to continue. The baseline characteristics of the 119 patients are described in Table 1.

**Table 1:** Baseline characteristics of participants (119).

Variables	Statistics
**Sociodemographics**	
**Gender, n (%)**	
Males	104 (87.4)
Females	15 (12.6)
**Age (years), mean (SD)**	66.9 (7.9)
**Educational Level, n (%)**	
Primary studies	84 (70.6)
More than primary	35 (29.4)
**Marital status, n (%)**	
Married	99 (83.2)
Without a partner or widower	20 (16.8)
**Frailty**	
**Frailty criteria, mean (SD)**	
Physical activity (METs)	2991 (3605.7)
Handgrip strength (Kg)	28.8 (7.5)
4MGS (m/s)	4.4 (1.0)
**Frailty status, n (%)**	
Non-frail	23 (19.3)
Pre-frail	87 (73.1)
Frail	9 (7.6)
**Clinical**	
**Pulmonary-related, mean (SD)**	
FEV_1_ (% predicted)	50.3 (16.5)
FVC (% predicted)	67.1 (19)
**Physical performance**	
**6WMT (meters), mean (SD)**	353.4 (85.2)
**SPPB score (range 0–12), mean (SD)**	9.7 (1.7)
**5STS test (in seconds), mean (SD)**	13.9 (3.0)
**5STS test, n (%)**	
≤ 13.6 seconds	57 (47.9)
> 13.6 seconds	62 (52.1)
**Quadriceps strength (Kg), mean (SD)**	15.9 (2.6)
**Self-perceived health**	
**BMI (kg/m**^**2**^**)**	29.1 (4.8)
**Smoking status, n (%)**	
Current smoker	36 (30.3)
Former smoker	83 (69.7)
**Comorbidities, n (%)**	
0–3	77 (64.8)
≥ 4	42 (35.2)
**Exacerbations**^**a**^**, n (%)**	
0–1	51 (42.9)
≥ 2	68 (57.1)
**Depression, n (%)**	
< 11 HAD-D	111 (93.3)
≥ 11 HAD-D	8 (6.7)
**Other health-related variables**	
**Dyspnea, n (%)**	
< 2 mMRC	81 (68.1)
≥2 mMRC	38 (31.9)
**CAT score, mean (SD)**	13.4 (6.7)
**CAT score, n (%)**	
< 10 points	34 (28.6)
≥ 10 points	85 (71.4)
**36-Short self-reported health, n (%)**	
Poor-very poor	70 (58.9)
Good to excellent	49 (41.1)
**Self-reported mobility, mean (SD)**	19.8 (16.1)

a: Moderate or severe exacerbations in the previous year

**Abbreviations:** SD, standard deviation; METs, metabolic equivalent task; 4MGS, 4-m gait-speed test; FEV_1,_ forced expiratory volume in 1s; FVC, forced vital capacity; 6MWT, six-minute walking test; SPPB, short physical performance battery; 5STS, five-times sit-to-stand; BMI, body mass index; HAD-D, Hospital and Anxiety Depression Scale (Depression subscale); mMRC, modified Medical Research Council; CAT, COPD assessment test.

The patients that missed the follow-up visits were not significantly different in most characteristics from those who participated throughout the study, including age (66.5 ±10.5 *vs* 66.9±7.9, respectively; *p* = 0.843), sex (88.8% *vs* 87.4% of males, respectively; *p* = 0.858), educational level (83.3% *vs* 70.5% of primary studies, respectively *p* = 0.0.281), %FEV1 (49.5±16.8 vs 50.3±16.5, respectively; *p* = 0.857) and self-reported health status (72.1% vs 58.9% of poor-very poor, respectively; *p* = 0.210). However, compared to patients who completed the study, those patients that missed their follow-up had more severe dyspnea (61.1% vs 31.9% of ≥ 2mMRC, respectively; *p* = 0.016) and higher CAT scores (18.9±8.9 vs 13.4±6.7, respectively; *p* = 0.003).

### Frailty transitions

At the end of the 2-year follow-up, 21 patients (17.7%) improved their frailty status (17 patients changed from pre- to non-frail and 4 from frail to pre-frail status), 14 patients (11.8%) worsened (5 pre-frail patients changed to frail status, 7 non-frail changed to pre-frail, and only 2 non-frail patients changed to frail status) and 84 patients (70.5%) did not change their initial frailty status.

**Fig 1:**
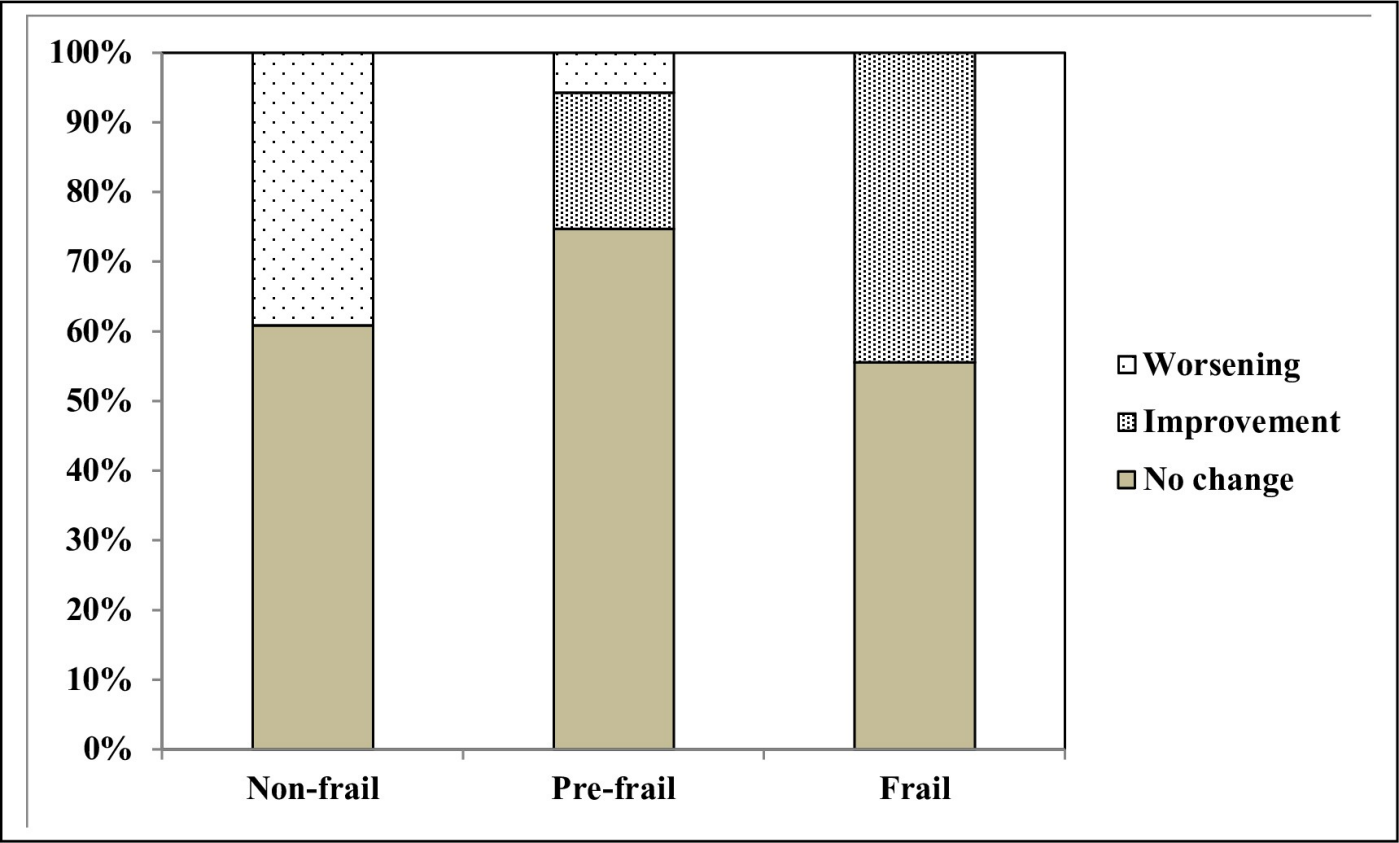
Frailty transitions according initial frailty status.

Fig 1 shows the patterns of changes (no change, improvement or worsening) according to the initial frailty status. The proportion of frail patients who improved their condition and non-frail patients who worsened was similar, approximately 40%. In the pre-frail group only 25% changed their initial status (19.5% improved and 5.7% worsened).

**Fig 2:**
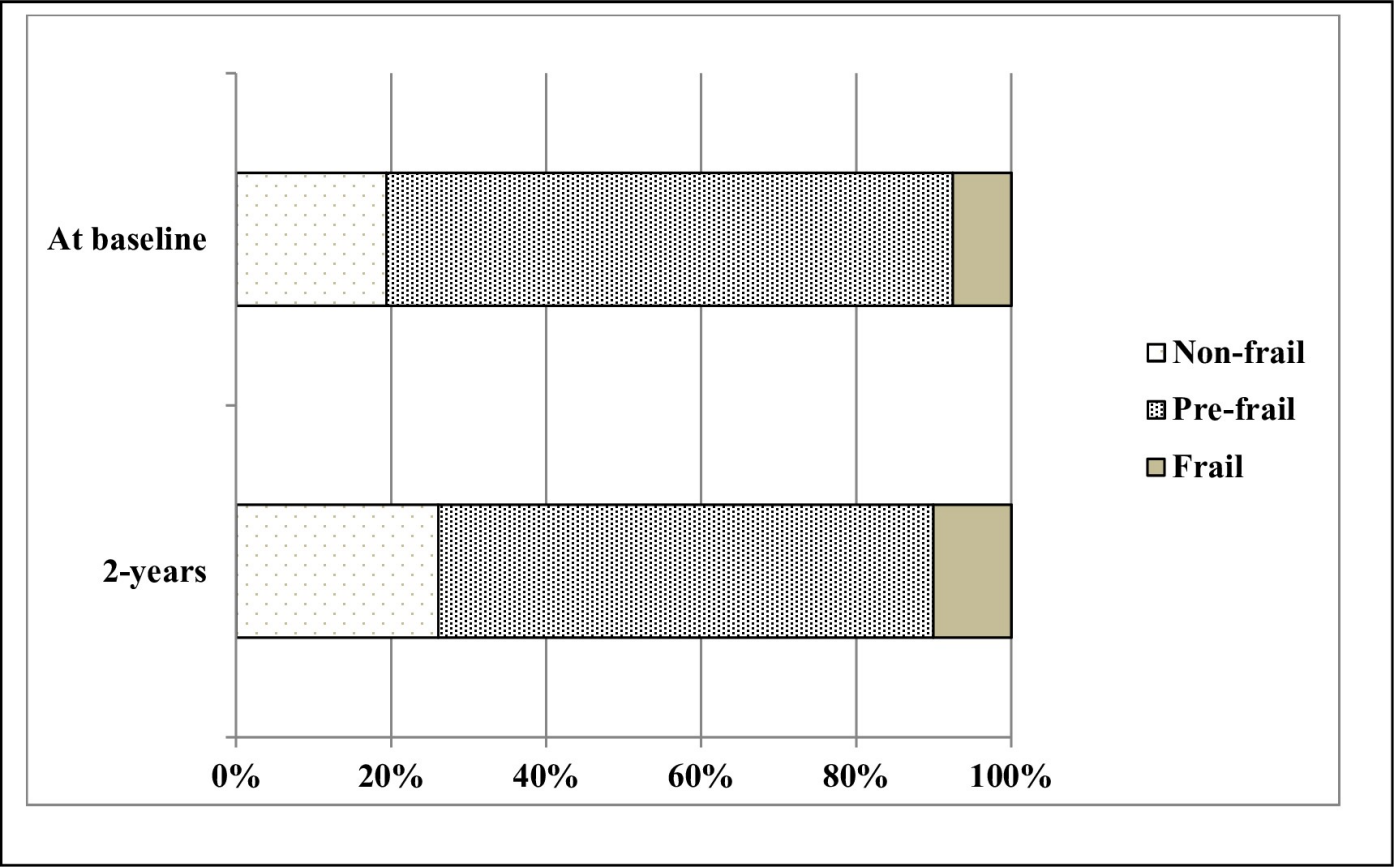
Frailty status at baseline and at the end of the 2 years of follow-up.

Fig 2 presents the proportion of patients by frailty status at baseline and 2 years. At the end of the 2-year follow-up, a total of 12 (10.1%) patients were frail, 76 (63.8%) pre-frail, and 31 (26.1%) non-frail. The proportion of pre-frail patients decreased with respect to baseline (73.1% vs 63.8%) and the proportion of non-frail and frail patients increased with respect to baseline (19.3% *vs* 26.1%; 7.6% *vs* 10.1%, respectively) (Fig 2).

### Clinical outcomes by frailty transition

Table 2 reports the mean change for clinical outcomes after 2 years of follow-up by patterns of change in frailty status (no change, improvement and worsening). The one-way analysis of variance (with Bonferroni post hoc) showed no significant differences at baseline between the groups in clinical outcome variables, except for physical activity (*p* = 0.040) and CAT score (*p* = 0.037) (Data not shown in the table). At baseline, the worsening group showed higher METs (*p* = 0.048) and CAT score (*p* = 0.032) than the improvement group (Data not shown in the table).

**Table 2:** Comparison of clinical outcomes following 2-years according to changes in frailty status (n = 119).

	Patterns of changes in frailty status			
Outcomes Variables	No change (n = 84)	Improvement (n = 21)	Worsening (n = 14)	*p* Value
**Pulmonary-related**				
FEV_1_ (% predicted)	2.03 (-0.24–4.32)	5.09 (-6.34–16.53)	4.21 (-1.40–9.83)	0.626
FVC (% predicted)	7.01 (4.18–9.83)	5.21 (-0.97–-11.39)	12.42 (-1.60–26.46)	0.346
**Physical performance**				
6MWT (meters)	-8.35 (-23.97–7.25)	19.84 (-21.13–60.81)	-24.30 (-80.09–31.47)	0.217
SPPB score (range 1–12)	0.08 (-0.26–0.43)	0.38 (-0.12–0.88)	-0.42 (-1.53–0.67)	0.333
5STS (in seconds)	-0.64 (-1.16 –-0.12)	-2.09 (-3.09 –-1.08)	-0.11 (-3.34–3.10)	0.068
Quadriceps strength (Kg)	1.43 (0.84–2.01)	3.18 (1.92–4.45)*	-0.01 (-1.88–1.85)†	0.003
**Self-perceived health**				
mMRC (0–4)	0.15 (-0.00–0.31)	0.28 (0.03–0.54)	0.78 (0.17–1.39)*	0.017
CAT score (range 0–40)	-0.04 (-1.36–1.26)	-0.85 (-3.77–2.06)	1.07 (-2.53–4.67)	0.662
Self-reported health (range 1–5)	-0.10 (-0.37–0.16)	0.57 (0.00–1.13)	-0.28 (-1.05–0.47)	0.062
Self-reported mobility (range 0–100)	0.31 (-3.26–3.89)	-3.49 (-9.86–2.88)	12.85 (-0.6–26.31)*†	0.017
**Frailty criteria**				
Physical activity (METs)	-591.40 (-1355.53–172.72)	1479.31 (-268.08–3226.7)	-2203.52 (-3777.32– -629.72)†	0.008
Handgrip strength (Kg)	-0.80 (-1.54 –-0.07)	3.00 (1.16–4.84)*	-4.61(-6.88 –-2.34)*†	<0.001
4MGS (m/s)	0.04 (-0.25–0.34)	-0.42 (-0.71 –-0.13)	0.49 (-0.65–1.65)	0.149

Values are mean change (IC95%) baseline to 2-years.

*Statistically different to “No change” group

† Statistically different to “Improvement” group

Abbreviations: FEV_1_, forced expiratory volume in 1s; FVC, forced vital capacity; 6MWT, six minute walking test; SPPB, short physical performance battery; 5STS, five-times sit-to-stand

mMRC, modified Medical Research Council; CAT, COPD assessment Tool; METs, metabolic equivalent task; 4MGS, 4-m gait-speed test.

We observed significant differences in the mean change across the groups for dyspnea, quadriceps, and disability, as well as for outcomes related to frailty characteristics (handgrip and physical activity) (Table 2). The Bonferroni post hoc test showed additional differences by pairs of groups. With respect to the no change group, the worsening group had a greater mean change and worsening of dyspnea (*p* = 0.013), disability (*p* = 0.036), and handgrip strength (*p* = 0.001). In contrast, the improvement group had a greater mean change and improvements of handgrip (*p*<0.001) and quadriceps strength (*p* = 0.032). In comparison with the worsening group, the improvement group showed greater handgrip strength (*p*<0.001), quadriceps strength (*p* = 0.003), physical activity (*p* = 0.008), and lower disability (*p* = 0.019).

### Predictors of improvement in frailty status vs. worsening

Table 3 shows Chi-square and Odds Ratio (OR) unadjusted according to covariates. A significantly greater proportion of the improvement group performed the 5STS test in less than 13.6 seconds compared with the worsening group (47.6% *vs* 7.1%, respectively, *p* = 0.012). In the worsening group there were higher percentages of patients with more exacerbations (85.7% *vs* 38.1%, respectively, *p* = 0.005) and higher CAT score (16.9 *vs* 10.9, respectively, *p* = 0.023) than in the improvement group. Five variables with *p*<0.20 (FEV_1_%, 5STS, CAT score, exacerbations and self-reported mobility) were candidates for the multivariate regression, but only 5STS (adjusted OR 9.46, 95% CI 0.92–96.83, *p* = 0.058) and exacerbations (adjusted OR 0.12, 95% CI 0.02–0.77, *p* = 0.026) were retained in the final model. These two variables explained 42% of the model. In this multivariate model the Chi-square value of the Hosmer-Lemeshow test was 0.325 with a significant level of 0.850, indicating support of the model (Table 3).

**Table 3:** Chi-square and Odds Ratio (Unadjusted) values of covariates between improvement and worsening groups (n = 35).

Variables	Improvement (n = 21)	Worsening (n = 14)	*p Value*	Unadjusted OR
	Statistics			(95%IC)
**Sociodemographics**				
Males, n (%)	19 (90.5)	11 (78.6)	0.324^b^	2.59 (0.37–17.97)
Age (years), mean (SD)	65.6 (6.3)	65.7 (10.2)	0.966^c^	0.99 (0.91–1.08)
Primary studies, n (%)	12 (57.1)	9 (64.3)	0.673^b^	0.74 (0.18–2.98)
Married, n (%)	18 (85.7)	13 (92.9)	0.515^b^	0.46 (0.04–4.95)
**Clinical**				
**Pulmonary-related**				
FEV_1_ (% predicted), mean (SD)	56.2 (21.9)	46.2 (9.9)	0.120^c^	1.03 (0.99–1.07)
FVC (% predicted), mean (SD)	72.9 (24.3)	65.7 (12.8)	0.319^c^	1.01 (0.98–1.05)
**Physical performance**				
6WMT (meters), mean (SD)	358.4 (108.8)	340.0 (88.3)	0.602^c^	1.00 (0.99–1.00)
5STS (≤ 13.6 seconds), n (%)	10 (47.6)	1 (7.1%)	0.012^b^	11.81 (1.30–107.39)
Quadriceps strength (Kg), mean (SD)	15.6 (2.8)	16.3 (2.7)	0.489^c^	0.91 (0.71–1.77)
**Self-perceived health**				
Dyspnea (≥ 2 mMRC), n (%)	4 (19.0)	4 (28.6)	0.511^b^	0.58 (0.12–2.88)
CAT score, mean (SD)	10.9 (6.1)	16.9 (8.7)	0.023^c^	0.89 (0.80–0.99)
36-Short self-reported health				
(good to excellent), n (%)	6 (28.6)	4 (28.6)	1.000^b^	1.00 (0.22–4.46)
Self-reported mobility, mean (SD)	14.7 (10.5)	23.3 (20.0)	0.107^c^	0.95 (0.90–1.01)
**Other health-related variables**				
BMI (kg/m^2^), mean (SD)	28.4 (3.9)	29.7 (4.6)	0.403^c^	0.93 (0.78–1.09)
Current smoker, n (%)	5 (23.8)	6 (42.9)	0.234^b^	0.41 (0.97–1.79)
Comorbidities (≥ 4), n (%)	6 (28.6)	3 (21.4)	0.636^b^	1.46 (0.29–7.18)
Exacerbations^a^ (≥ 2), n (%)	8 (38.1)	12 (85.7)	0.005^b^	0.10 (0.01–0.58)

a: Moderate or severe exacerbations in the previous year

b: p value for Chi-square test

c: p value for *t*-test.

**Abbreviations:** SD, standard deviation; FEV1, forced expiratory volume in 1s; FVC, forced vital capacity; 6MWT, six-minute walking test; 5STS, five-times sit-to-stand; mMRC, modified Medical Research Council; CAT: COPD assessment test; BMI: body mass index.

## Discussion

In this study, one-third of the stable COPD patients changed their frailty status after 2 years of medical follow-up. We observed that the transitions in frailty status were also associated with significant clinical outcomes related to dyspnea, mobility, physical activity, and handgrip and quadriceps strength. In addition, the 5STS and exacerbations were independent predictors of improvement in frailty status.

The transition rates for improvement and worsening over the total cohort in this study were slightly different from the current literature for the geriatric population. Our patients with COPD had a higher rate of improvement than community-dwelling older adults [12]. One possible reason for this difference is the inclusion of our patients in a systematic and annual medical visits program.

Regarding the subgroup analysis, our improvement rate for “frail patients” was lower than that reported for COPD patients in a pulmonary rehabilitation setting (61% vs. 43%, respectively) [15]. This is expected because these programs are highly effective at improving many components of frailty, including slowness, fatigue, weakness, and physical activity. In addition, some programs include other frailty-related outcomes, such as balance training.

We corroborated the results of previous studies reporting that frailty transitions are more frequent between adjacent states (one-step transitions) [10–12]. The rare two-step transition in our study was a worsening from non-frail to frail status. This unexpected result can be explained by the severity of these two patients at baseline (data not reported).

Important clinical outcomes were associated with frailty transitions at the end of 2 years in both the worsening and improvement groups. Improved frailty status was associated with increased peripheral muscle strength (handgrip and quadriceps strength), whereas worsening frailty was associated with worsening dyspnea, mobility, and handgrip strength. These changes are clinically relevant, as they exceed the minimal important difference (MID) considered elsewhere as the 0.5 standard deviation of scores at baseline (unexposed data) [26]. We cannot corroborate the results reported for patients in a rehabilitation setting because they only examine clinical outcomes associated with frailty status at the end of the follow-up, not those associated with transitions from baseline. To the best of our knowledge, this is the first study exploring changes in clinical outcomes according to changes in the frailty status of COPD patients.

In this study, 5STS and the number of exacerbations (moderate or severe) were found to be independent predictors of improvement (vs. worsening) of the initial frailty status. It was not surprising that the number of exacerbations could predict improvement in frailty status, as its role in clinical outcomes (i.e., mortality) of COPD is widely recognized. Some factors, such as greater leg power, being married, and good or excellent self-reported health, have been associated with frailty improvement in other patient populations [10]. For our patients with COPD, marital status was not associated with frailty improvement, and health status as measured by CAT was only related in the univariate analysis. On the other hand, although the 5STS test is partly dependent on lower limb muscle, the quadriceps strength was not associated with frailty improvement. Thus, it seems that the 5STS, as a measure of functional performance, captures the multisystemic effects of COPD in a more integrated way [27]. Previous studies have demonstrated the ability of the 5STS to identify COPD patients with poor exercise tolerance and risk of disability [21, 28]. Our study adds to the literature that 5STS is also a predictor of improvement in frailty status.

### Implications for practice

Our study provides useful information for health policy-makers and health care professionals in the management of frailty in patients with COPD. On the one hand, because most patients did not change their initial frailty status after 2 years of follow-up, our results suggest that there is a window during which interventions can be applied to reduce or improve their frailty status. On the other hand, among the patients who experienced transition, the group of initially frail patients had a high rate of improvement, demonstrating that individuals classified as frail are not necessarily in an extreme and irreversible health condition. Multidisciplinary programs that include exercise, nutritional support, self-management strategies, and reduction polypharmacy, such as pulmonary rehabilitation, have been shown to be effective in reducing frailty [1, 15]. Finally, we think that our main contribution was to demonstrate that changes in frailty status are associated with changes in relevant outcomes for COPD patients, such as dyspnea or disability, and that the 5STS can be used as a simple bedside or clinical assessment to identify patients susceptible to improvements in frailty.

### Strengths and limitations of study

Our study has several strengths. First, we used a standardized and validated frailty measure widely used in other longitudinal studies. This will facilitate comparisons with future studies in patients with COPD, even in other contexts. Second, we have included in the analyses important clinical outcome variables for COPD patients (i.e., FEV_1_ or dyspnea) and several other variables previously identified in the literature as factors related to frailty. Finally, our follow-up period was long enough for patients to have changes in their frailty status.

Despite these strengths, our study also has limitations that should be highlighted. First, although we have used a standardized and validated frailty measure, as we have commented previously, the application of the same cut-off points described by Fried for another population might underestimate or overestimate the characteristics of this study population. Second, measurements were made only at two time points (baseline and 2 years), and it is likely that additional transitions occurred between these time points. Third, death at follow-up was not included as a worsening frailty transition, and this might have affected our final results, especially for pre-frail and frail patients who were at higher risk of mortality than the non-frail patients. In addition, as the analysis of frailty transitions was conducted concurrently with changes in clinical outcomes, it is not possible to determine that frailty transitions contributed to changes in clinical outcomes as opposed to the reverse (i.e., decreased disability improved frailty status).

## Conclusions

The results of the current study provide evidence that frailty is a dynamic process for approximately one-third of patients with stable COPD and important clinical outcomes are associated with frailty transitions. Improvement in frailty status was associated with increased peripheral muscle strength (handgrip and quadriceps strength), whereas worsening frailty was associated with worsening dyspnea, mobility, and handgrip strength. In addition, we have shown that the 5STS and exacerbations are independent predictors of improvement in frailty status.

## Supporting information

S1 Data (XLSX)
